# Psychometric properties of the Arabic versions of the Three-Item Short Form of the modified Weight Bias Internalization Scale (WBIS-3) and the Muscularity Bias Internalization Scale (MBIS)

**DOI:** 10.1186/s40337-023-00805-z

**Published:** 2023-05-23

**Authors:** Feten Fekih-Romdhane, Jinbo He, Diana Malaeb, Mariam Dabbous, Rabih Hallit, Sahar Obeid, Souheil Hallit

**Affiliations:** 1 The Tunisian Center of Early Intervention in Psychosis, Department of Psychiatry “Ibn Omrane”, Razi Hospital, Manouba City, Tunisia; 2 Faculty of Medicine of Tunis, Tunis El Manar University, Tunis, Tunisia; 3 School of Humanities and Social Science, The Chinese University of Hong Kong, Shenzhen, 518172 Guangdong China; 4 College of Pharmacy, Medical Gulf University, Ajman, United Arab Emirates; 5 School of Pharmacy, Lebanese International University, Beirut, Lebanon; 6 School of Medicine and Medical Sciences, Holy Spirit University of Kaslik, Jounieh, Lebanon; 7 Department of Infectious Disease, Bellevue Medical Center, Mansourieh, Lebanon; 8 Department of Infectious Disease, Notre Dame des Secours University Hospital, Postal Code 3 Byblos, Lebanon; 9 Social and Education Sciences Department, School of Arts and Sciences, Lebanese American University, Jbeil, Lebanon; 10 Psychology Department, College of Humanities, Effat University, Jeddah, 21478 Saudi Arabia; 11 Applied Science Research Center, Applied Science Private University, Amman, Jordan; 12 Research Department, Psychiatric Hospital of the Cross, Jal Eddib, Lebanon

**Keywords:** Weight Bias Internalization Scale, Muscularity Bias Internalization Scale, Internalized bias, Muscularity bias, Arabic, Psychometric properties

## Abstract

**Background:**

There is a lack of psychometrically sound measures to assess internalized weight and muscularity biases among Arabic-speaking people. To fill this gap, we sought to investigate the psychometric properties of Arabic translations of the Three-Item Short Form of the Modified Weight Bias Internalization Scale (WBIS-3) and the Muscularity Bias Internalization Scale (MBIS) in a sample of community adults.

**Methods:**

A total of 402 Lebanese citizens and residents enrolled in this cross-sectional study (mean age: 24.46 years (*SD* = 6.60); 55.2% females). Exploratory Factor Analysis (EFA) was conducted using the principal-axis factoring and oblimin rotation to estimate parameters and the parallel analysis to determine the number of factors. CFA was conducted using the weighted least square mean and variance adjusted estimator which was recommended for ordinal CFA.

**Results:**

An Exploratory Factor Analysis of the WBIS-3 resulted in a robust single-factor solution for the three items. An examination of the factorial structure of the MBIS revealed a two-factor structure, which showed adequate model fit. We obtained excellent internal consistency as indicated by McDonald’s ω coefficients of .87 for the WBIS-3 total score and ranging between .92 and .95 for the MBIS two factor scores. Cross-sex invariance of the MBIS was confirmed at the configural, metric, and scalar levels. Convergent validity was supported by significant correlations between the WBIS-3 and MBIS. Divergent and concurrent validity were approved by showing small to medium correlations between MBIS/WBIS-3 scores and muscle dysmorphia, disordered eating symptoms, and body image concerns.

**Conclusion:**

Findings suggest that the Arabic versions of the WBIS-3 and MBIS are suitable for use in Arabic-speaking adults.

## Abstract (plain language summary)

Individuals with overweight or obesity frequently experience weight-based stigmatization. The widespread belief that weight is a matter of personal will and self-control results in various weight-based stereotypes (e.g., laziness, lack of self-discipline, or neglect). Furthermore, the influence model of body image identifies internalization of societal body ideals as a risk factor for developing body dissatisfaction, and subsequent disordered eating behavior. Hence, internalization of two dimensions of body image ideals, thinness and muscularity, is associated with body dissatisfaction and eating concerns. Weight Bias Internalization Scale (WBIS-3) is a shortened version of the modified version of the Weight Bias Internalization Scale (WBIS-M) designed to evaluate the weight bias internalization. As for the Muscularity Bias Internalization Scale (MBIS), it assesses the extent to which an individual endorses muscularity-based stereotypes and engages in negative self-evaluations due to muscularity. These two scales are not yet validated in Arabic. To that end, we aimed to translate and validate the Arabic version of the WBIS-3 and MBIS in the present study, which would in turn facilitate improved research and clinical practices related to weight and muscularity biases internalization in Arabic-speaking nations. The present findings provide support for the psychometric properties of the Arabic version of the WBI-3 and MBIS in examining weight and muscularity biases internalization in Arabic-speaking adults in Lebanon.

## Introduction

Weight stigma, also referred to as weight bias, is a prominent health concern globally [1]. It can be defined as social denigration and devaluation of an individual due to their body weight, often leading to negative anti-fat attitudes and stereotypes [2]. Weight bias internalization (WBI) differs from weight stigma, as in WBI, the attribution is made towards the “self”, not towards the “other” [3]. In other words, the person internalizes society’s negative weight stereotypes, apply them to him/herself; and, therefore, devalues themselves/their self-worth because of their weight [3–5]. WBI is also a distinct construct from body image [6], but individuals who internalize weight bias may see themselves as unattractive or feel guilty due to their weight [7]. Even though WBI was initially thought to only affect specific groups with overweight/obesity, recent research has shown that individuals can experience WBI regardless of their weight status [8–10]. In addition to experiencing WBI, individuals may also internalize muscularity-based stereotypes and subsequent self-devaluation because of one’s muscularity, which is referred to as muscularity bias internalization (MBI) [11]. MBI is different from drive for muscularity, as the latter involves attitudes and behaviors reflecting one’s desire to have a muscular body [12], whereas MBI occurs when the person engages in negative self-evaluations and internalizes negative muscle-based beliefs because of their muscle mass [11]. Therefore, MBI has been proposed as “a precursor of drive for muscularity” [11], with individuals exhibiting higher MBI being expected to also have higher drive for muscularity.

Both WBI and MBI are relatively new concepts in weight and muscle discrimination research areas; and they are gaining growing attention from clinicians and researchers in recent years because of their clinical relevance and potential implications for public health. Indeed, WBI has been consistently associated with decreased physical activity [13], body image issues [3, 7], and disordered eating behaviors [14], which may, in turn, perpetuate obesity and hinder weight-loss maintenance [15, 16]. WBI has also been found to be linked to poor physical health [17, 18], as well as negative psychological consequences, including anxiety, stress, depression, negative affect, maladaptive coping responses, and low self-esteem [3, 5, 7, 14, 19, 20]. Similarly, MBI was demonstrated to correlate with a range of negative mental health outcomes, such as psychosocial impairment, more body dissatisfaction, less body appreciation, thinness-oriented disordered eating symptoms, and muscle dysmorphic disorder symptoms [11]. In addition, MBI was found to significantly correlate with muscularity-oriented disordered eating symptoms, above and beyond the effects of drive for muscularity [11]. Overall, both WBI and MBI seem to be pervasive and substantially detrimental for health. Given the existence of some evidence supporting the effectiveness of interventions for WBI (e.g., [21]) and MBI (e.g., a study targeted irrational beliefs about muscularity [22]) in reducing weight- and muscularity-oriented body image concerns and disordered eating, these constructs should be routinely assessed and addressed.

One of the most frequently used measures of WBI is the 11-item Weight Bias Internalization Scale (WBIS) [3]. The scale was initially designed with the purpose of measuring WBI in individuals with overweight or obesity. However, given the consistent evidence that individuals with normal-weight (those with adequate body mass index (18.5–24.9 kg/m^2^) also experience WBI [23], a modified version of the WBIS (The WBIS-M; [24]) was developed to adapt the scale for use across various body weight statuses. The WBIS-M has been translated and adapted to various cultures and languages over the recent years, including Spanish [25], Greek [26], Chinese [27], and Turkish [28]. More recently, a shortened 3-item version of the WBIS-M (i.e., WBIS-3) has been validated in the German general population [29]. The WBIS-3 was found equivalent to the full version with a correlation of *r* = 0.94 [29]. In addition to its demonstrated excellent psychometric properties (including good internal consistency, appropriate construct validity, and strong measurement invariance) [29], the WBIS-3 offers potential advantages in terms of easiness/rapidity of administration, low cost, and less burden to respondents. On the other hand, specific measures focusing on MBI were until recently nonexistent; with only one instrument available to assess the construct of muscular-ideal internalization (i.e. the Sociocultural Attitudes Towards Appearance Questionnaire‐4–Revised [SATAQ‐4R]; [30]). In 2022, He et al. developed a new measure of MBI, the Muscularity Bias Internalization Scale (MBIS; [11]); which is composed 14 items and three-factors. The MBIS showed adequate psychometric qualities in Chinese adult men in terms of internal consistency (categorical ω values ranging from 0.76 to 0.99 for the total score and all three sub-scores), construct validity and test–retest reliability. To the best of our knowledge, no measures are available in the Arabic language to assess the WBI and MBI constructs in Arabic-speaking populations. As such, no epidemiological prevalence data regarding WBI and MBI are available to date for Arab countries and the broader Arabic-speaking communities.

Prevalence rates of obesity and its related diseases have been steadily rising over the past decades and have become a major public health problem worldwide [1], with Arab countries being of no exception [31]. With regard to Arab countries, the situation is even more critical due to the close relationship between food and people’s identity/culture/traditions, sociocultural-related barriers to physical activity practice, as well as a widespread concerning lack of public awareness [31, 32]. Additionally, due to globalization, Arab people are increasingly adopting Western cultural and social beliefs and practices [33]; with a substantial rise in the internalization of thin and muscularity ideals [34]. For instance, there is some evidence that body dissatisfaction is among the strongest correlate of eating disorder pathology and an array of other negative psychological indicators in Arab populations (e.g., Saudi students [35]; Lebanese adults [36, 37] and adolescents [38]; Jordanian adolescent schoolgirls [39]; Emirati adolescents [40]; Bahraini adolescents [41]). A study by O’Hara et al. [42] showed that 44% of female Emirati undergraduate students reported being frequently teased about their weight; and that internalized weight stigma was the most powerful predictor of eating disorder symptomatology. In light of their findings, authors suggested that these issues should be considered as priorities for action by public health authorities [42]. Despite these facts, most of the limited existing research on eating disorders symptomatology, body image disturbances and related problems emerging from the Arab world has traditionally focused on females [43], and has long suffered from a lack of validated instruments [34]. The few scales available are thinness-oriented (e.g., [44–46]); and it is only recently that a muscularity-oriented scale has been validated (i.e., [47]). No scales on weight- or muscularity-related stigmatizing beliefs exist to our knowledge. The vast majority of data on this topic originated from the United States [48], and other Western countries [49–51]. Global studies with cross-cultural comparisons remain scarce [52–54], in spite of being identified as a key future research direction [14]. This highlights the strong need for making measures assessing WBI and MBI available for Arabic-speaking people in all parts of the world. To this end, we sought through the present paper to investigate the psychometric properties of Arabic translations of the WBIS-3 and the MBIS in a sample of Arabic-speaking community adults from Lebanon. We hypothesized that both scales would show excellent psychometric properties in their Arabic versions. In particular, we expect that the originally proposed single-factor structure of the WBIS-3 and three-factor structure of the MBIS would be confirmed in our sample. In addition, we expect that MBIS scores would be invariant across sex groups. We also expect that the two scales would show good composite internal consistency (McDonald’s ω values greater than 0.70 for WBIS-3/MBIS total scores and MBIS sub-scores), and that good convergent, divergent and concurrent validity will be evidenced through adequate patterns of correlations of both WBI and MBI with body appreciation, disordered eating and muscle dysmorphic symptoms.

## Methods

### Procedures

In this cross-sectional study that involved a convenience community sample, all data were collected via a Google Form link, between December 2022 and January 2023. The project was advertised on social media and included an estimated duration. Inclusion criteria for participation included being of a resident and citizen of Lebanon of adult age. Internet protocol (IP) addresses were examined to ensure that no participant took the survey more than once. After providing digital informed consent, participants were asked to complete the instruments described above, which were presented in a pre-randomized order to control for order effects. The survey was anonymous and participants completed the survey voluntarily and without remuneration.

### Measures

#### Muscle Bias Internalization Scale (MBIS)

This scale is composed of 14 items scored on a 7-point Likert Scale (“1 = Strongly disagree to ‘7 = Strongly agree”). Higher scores indicate higher levels of muscularity bias internalization [11]. Personal Value attached to Muscularity (PVM), Perceived Impact of Muscularity (PIM), and Definition and Appearance of Muscularity (DAM). The MBIS showed a three-factor structure (Personal Value attached to Muscularity, Perceived Impact of Muscularity, and Definition and Appearance of Muscularity) and good reliability and validity in Chinese adult men, with a 0.90 Cronbach’s alpha [11].

#### Weight Bias Internalization Scale (WBIS-3)

This scale is a shortened version of the modified version of the Weight Bias Internalization Scale (WBIS-M) [24], exhibiting excellent psychometric properties with an internal consistency of α = 0.92 [29]. The WBIS-3 is composed of the following three items: “I hate myself for my weight”, “Whenever I think a lot about my weight, I feel depressed”, and “I feel anxious about my weight because of what people might think of me”. Each item is scored on a 7-point Likert Scale ranging from 1 (Strongly disagree) to 7 (Strongly agree). Higher scores indicate higher levels of weight-related self-stigma.

#### Body Appreciation Scale-2 (BAS-2)

Validated in Arabic [55], this 10-item instrument assesses acceptance of one’s body, respect and care for one’s body, and protection of one’s body from unrealistic beauty standards. Previous research found a unidimensional factor structure, along with strong internal consistency (Cronbach’s α = 0.97), construct validity and test–retest reliability (r = 0.90) in community and college samples of men and women [56]. All items were rated on a 5-point scale, ranging from 1 (*never*) to 5 (*always*) [56]. Higher scores on this scale reflect greater body appreciation. McDonald’s ω was 0.97/Cronbach’s α = 0.97 in the total sample.

#### Eating Attitudes Test-7 (EAT-7)

Participants were asked to complete the EAT-7 which has recently been validated in Arabic, with a one-factor solution, and an excellent Cronbach’s alpha (> 0.9) [57]. This 7-item scale measures symptoms and concerns characteristic of eating disorders. All items were rated on a 6-point scale, ranging from 1 (*never*) to 6 (*always*). Higher total scores reflect greater disordered eating attitudes. In the present study, McDonald’s ω was 0.80/Cronbach’s α = 0.80 in the total sample.

#### Muscle Dysmorphic Disorder Inventory (Ar-MDDI)

Validated in the Arabic language [47], the results of the EFA revealed three factors (Appearance intolerance, Drive for size, and Functional impairment) with a Cronbach’s alpha of 0.81. This scale is composed of 13 items, scored on a five-point Likert-type scale (0 = never to 4 = always) [58]. In the present study, McDonald’s ω was 0.88/Cronbach’s α = 0.90 in the total sample.

#### Demographics

Participants were asked to provide their demographic details consisting of age, sex, marital status, highest education level, and physical activity (calculated by multiplying the exercise strength by intensity by duration [59]).

#### Translation procedure

The WBIS-3 and MBIS scales were translated to the official Arabic language, which is written and spoken across the Middle East and North Africa (MENA). The translation was performed with the purpose of achieving semantic equivalence between measures in their original and Arabic versions following international norms and recommendations [60]. To this end, the forward–backward translation approach was used. The English version was translated to Arabic by a Lebanese translator who was completely unrelated to the study. Afterwards, a Lebanese psychologist with a full working proficiency in English, translated the Arabic version back to English. The translation team ensured that any literal and/or specific translation was balanced. The initial and translated English versions were compared to detect/eliminate any inconsistencies and guarantee the accuracy of the translation by a committee of experts composed of the research team and the two translators [61]. An adaptation of the measure to the Arab context was performed, and sought to determine any misunderstanding of the items wording as well as the ease of items interpretation; and, therefore, ensure the conceptual equivalence of the original and Arabic scales in both contexts [62]. After the translation and adaptation of the scale, a pilot study was done on 20 participants to ensure all questions were well understood; no changes were applied after the pilot study.

### Analytic strategy

#### Data treatment

There were no missing responses in the dataset. To examine the factor structure of the MBIS, we used an EFA-to-CFA strategy [63]. To ensure adequate sample sizes for both EFA and CFA (i.e., n = 201 for EFA and CFA), we split the main sample using an SPSS computer-generated random technique; sample characteristics of the two split-halves are reported in Table 1. To examine the factor structure of the WBIS-3, we use EFA only, since CFA with three items is a saturated model.

**Table 1:** Sociodemographic characteristics of the participants

Variable	First split-half subsample (*n* = 201)	Second split-half subsample *(n* = 201)	χ^2^/t	*p*
Sex			.362	.547
Male	87 (48.3%)	93 (51.7%)		
Female	114 (51.4%)	108 (48.6%)		
Marital status			1.770	.183
Single	162 (48.5%)	172 (51.5%)		
Married	39 (57.4%)	29 (42.6%)		
Education			.225	.635
Secondary or less	24 (53.3%)	21 (46.7%)		
University	177 (49.6%)	180 (50.4%)		
	Mean ± SD	Mean ± SD		
Age (in years)	25.02 (6.83)	23.90 (6.33)	1.711	.088
Physical activity	25.91 (18.90)	25.23 (20.44)	.345	.731
Muscle dysmorphic disorder	2.14 (.86)	1.94 (.70)	2.506	**.013**
Body appreciation	3.74 (1.01)	3.64 (1.12)	.944	.346
Eating attitudes	.32 (.53)	.26 (.39)	1.187	.236

Numbers in bold indicate significant *p* values

Numbers are shown as mean (standard deviation) or frequency (percentage)

#### Exploratory Factor Analysis

EFA was conducted via the psych package [64]. We used parallel analysis to determine the number of factors. We used principal-axis factoring and oblimin rotation to estimate parameters in EFA.

#### Confirmatory Factor Analysis

CFA was conducted via the lavaan package [65]. Given that the responses for the MBIS are ordinal (i.e., Likert scales), we used the weighted least square mean and variance adjusted (WLSMV) estimator which was recommended for ordinal CFA [66]. Following the guidelines in Hu and Benlter [67], the following model fit indicators were used, the Comparative Fit Index (CFI; values close to or greater than 0.95 = good fit), the Tucker-Lewis index (TLI; values close to or greater than 0.95 = good fit), and Standardized Root Mean Square Residual (SRMR; values close to or less than 0.05 = good fit, and values between 0.06 and 0.10 = acceptable fit). Note that SRMR performed generally better than RMSEA in CFA with ordinal data [68], we did not report RMSEA in the present study.

#### Measurement invariance

To examine sex invariance of MBIS scores, we conducted multi-group CFA [69] using SPSS AMOS v.29 software on the second split-half subsample with the estimator of ML. We did not use the WLSMV is because WLSMV requires the two groups to have the same missing pattern in all categories; however, there were certain items that had no values in certain categories (e.g., 7 “strongly agree”) for female. Thus, WLSMV was not applicable. Measurement invariance was assessed at the configural, metric, and scalar levels [70]. Proof of invariance was estimated if ΔCFI ≤ 0.010 and ΔRMSEA ≤ 0.015 or ΔSRMR ≤ 0.010 [69, 71].

#### Further analyses

Composite internal consistency in both subsamples was assessed using McDonald’s ω (and Cronbach’s α), with values > 0.70 reflecting adequate internal consistency [72]. McDonald’s ω was selected as a measure of composite reliability because of known problems with the use of Cronbach’s α [73]. To assess convergent and criterion-related validity, we examined bivariate correlations between MBIS and WBIS scores and all scales included in the survey using the total sample. All scores had normal distribution, as identified by skewness and kurtosis values varying between − 1 and + 1 [74]; therefore, Pearson correlation test was used. Based on [75], values ≤ 0.10 were considered weak, ~ 0.30 were considered moderate, and ~ 0.50 were considered strong correlations. P < 0.05 was considered statistically significant.

## Results

A total of 402 Lebanese citizens and residents enrolled in this study with a mean age of 24.46 years (*SD* = 6.60; min = 18; max = 60) and 55.2% females. Other sample characteristics are displayed in Table 1. No significant differences were seen between the two subsamples in terms of all characteristics, except for the MDD score were participants from subsample 1 scored higher than those from subsample 2.

### Muscularity Bias Internalization Scale

#### Exploratory Factor Analysis

**Fig. 1:**
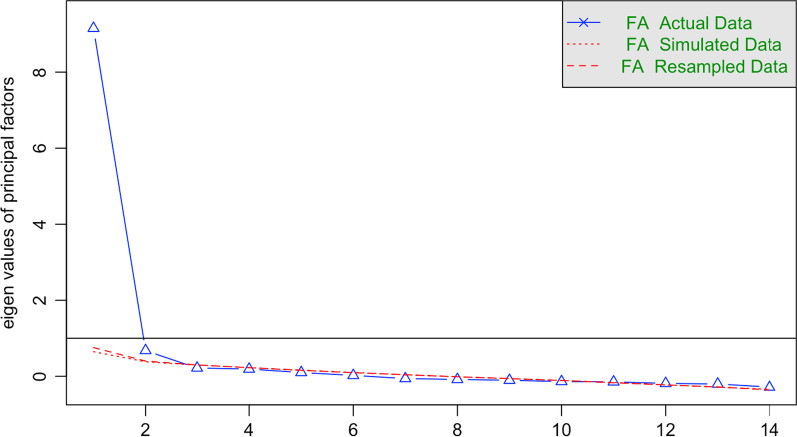
Parallel analysis of the MBIS

According to the results of Bartlett’s test of sphericity, *χ*^2^(91) = 2837.45 (p < 0.001), and the Kaiser–Meyer–Olkin Measure of Sampling Adequacy of 0.93, the items of the MBIS items were suitable for factor analysis. Parallel analysis (see Fig. 1) showed that two factors should be extracted. The two-factor solution could explain 71% of the total variance. The standardized factor loadings are shown in Table 2. The McDonald’s ω values were 0.95 for Factor 1 and 0.93 for Factor 2 in the first subsample (Cronbach’s α = 0.92 and 0.94 respectively).

**Table 2:** Items of the muscle and weight bias internalization scales in English and factor loadings derived from the Exploratory Factor Analyses (EFA) in the first split-half subsample, and standardised estimates of factor loadings from the Confirmatory Factor Analysis (CFA) in the second split-half subsample

	EFA	CFA
*Muscle Bias Internalization Scale*		
Factor 1: Personal value attached to muscularity		
MBIS 1	.58	.78
MBIS 2	.78	.87
MBIS 3	.65	.87
MBIS 4	.97	.91
MBIS 5	.89	.90
MBIS 6	.81	.95
Factor 2: Definition, appearance, and perceived impact of muscularity		
MBIS 7	.66	.91
MBIS 8	.72	.89
MBIS 9	.77	.89
MBIS 10	.61	.91
MBIS 11	.51	.89
MBIS 12	.82	.84
MBIS 13	.60	.89
MBIS 14	.96	.76

#### Confirmatory Factor Analysis

Results of CFA showed that the two-factor model had an adequate model fit, with *χ*^2^(76) = 627.58 (*p* < 0.001), CFI = 0.95, TLI = 0.95, and SRMR = 0.06. The two factors had a correlation of r = 0.87 (*p* < 0.001). The factor loadings are shown in Table 2. The McDonald’s ω values were 0.94 for Factor 1 and 0.92 for Factor 2 in the second subsample (Cronbach’s α = 0.94 and 0.95 respectively).

### Weight Bias Internalization Scale-3

#### Exploratory Factor Analysis

**Fig. 2:**
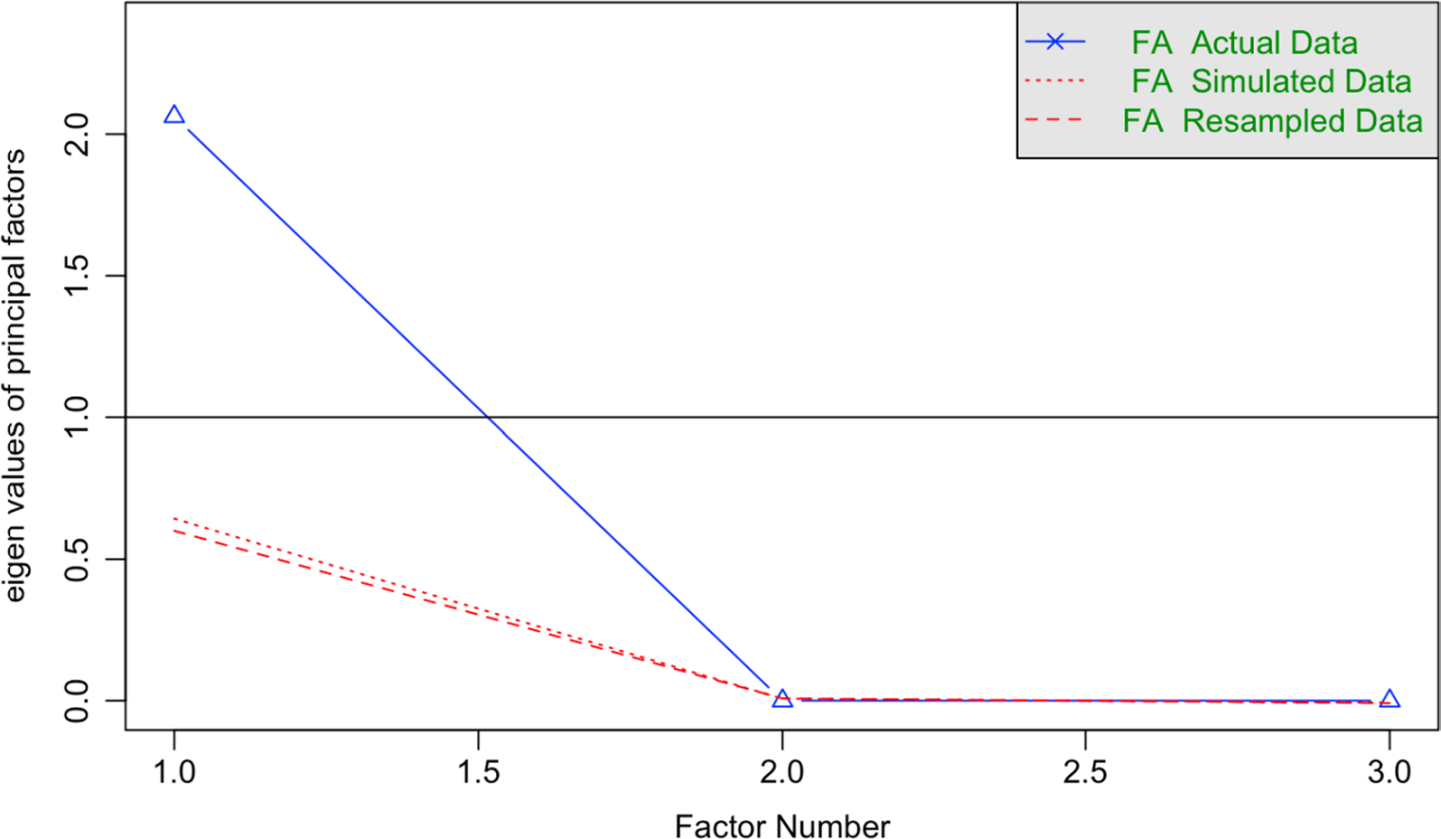
Parallel analysis of the WBIS-3

According to the results of Bartlett’s test of sphericity, *χ*^2^(3) = 587.24 (p < 0.001), and the Kaiser–Meyer–Olkin Measure of Sampling Adequacy of 0.73, the items of the WBIS-3 items were suitable for factor analysis. Parallel analysis (see Fig. 2) showed that one factor should be extracted, explaining 69% of the total variance. The standardized factor loadings are shown in Table 3. The McDonald’s ω values were 0.87 in the total sample, 0.86 in males and 0.88 in females (Cronbach’s α = 0.87 in the total sample, 0.86 in males and 0.87 in females).

**Table 3:** Loading factors deriving from the Exploratory Factor Analysis of the Weight Bias Internalization-3 items in the total sample and among males and females respectively

	Total sample	Males	Females
WBIS-4	.89	.87	.90
WBIS-2	.81	.78	.83
WBIS-6	.79	.80	.78

##### Measurement invariance

All fit indices suggested that measurement invariance was verified across sexes concerning the MBIS scale (Table 4). Higher mean MBIS Factor 1 and Factor 2 were significantly found in males compared to females. However, no difference was found between sexes in terms of WBIS scores (Table 5).

**Table 4:** Measurement invariance of the Muscle Bias Internalization Scale (MBIS) across sex in the second split-half subsample

Model	χ^2^	*df*	CFI	RMSEA	SRMR	Model comparison	Δχ^2^	ΔCFI	ΔRMSEA	ΔSRMR	Δ*df*	*p*
Configural	519.38	152	.875	.110	.066							
Metric	528.84	164	.876	.106	.066	Configural vs metric	9.46	.001	.004	< .001	12	.663
Scalar	539.33	177	.877	.101	.067	Metric vs scalar	10.49	.001	.005	.001	13	.653

We were unable to use the WLSMV to test the invariance of the MBIS, as there were no response categories in certain items for the male and/or female samples. Thus, for invariance test purpose, we used the ML estimator using SPSS AMOS v.29

*CFI* comparative fit index, *RMSEA* Steiger–Lind root mean square error of approximation, *SRMR* standardised root mean square residual

**Table 5:** Comparison between sexes in terms of muscle and weight bias internalization scores in the second split-half subsample

	MBIS Factor 1	MBIS Factor 2	WBIS-3
Males	15.63 (8.29)	23.66 (11.15)	8.28 (4.22)
Females	11.86 (6.36)	17.62 (10.27)	7.62 (4.48)
*p*	** < .001**	** < .001**	.129
Effect size	.510	.563	.151

Numbers in bold indicate significant *p* values. Numbers are shown as mean (standard deviation)

##### Convergent, divergent and concurrent validity in the total sample

Convergent validity was demonstrated by the correlation between the MBIS and WBIS, (r = 0.49, p < 0.001). Divergent validity was demonstrated by the correlation between the MBIS and the EAT (r = 0.14, p = 0.006), as well as by the significant correlation between the correlations of MBIS and MDDI (r = 0.51, p < 0.001) and WBIS and MDDI (r = 0.38, p < 0.001). Concurrent validity, demonstrated by the correlations between the MBIS and MDDI (r = 0.51, p < 0.001), between the MBIS and DMS (r = 0.61, p < 0.001), between MBIS and BAS-2 (r = 0.30, p <  − 0.001) (Table 6).

**Table 6:** Correlation matrix of continuous variables

	MBIS	WBIS	MDDI	BAS	EAT
1. MBIS	1				
2. WBIS	.49***	1			
3. MDDI	.51***	.38***	1		
4. BAS	− .30***	− .23***	− .38***	1	
5. EAT	.14**	.38***	.24***	− .004	1

Numbers refer to Pearson correlation coefficients

*MBIS* Muscularity Bias Internalization Scale, *WBIS* Weight Bias Internalization Scale, *MDDI* Muscle Dysmorphic Disorder Inventory, *BAS* Body Appreciation Scale-2, *EAT* Eating Attitude Test 7 items

^**^p < .01; ***p < .001

## Discussion

We sought to contribute to the underdeveloped area of research on WBI and MBI, by translating and validating the Arabic versions of the WBIS-3 and the MBIS for use in Arabic-speaking populations. Overall, the results confirmed good psychometric qualities of the Arabic translation of both scales. After performing both a CFA and an EFA of the MBIS using two different subsamples, the findings showed adequate fit of the 14-item Arabic MBIS version that fall into two factors, as well as a high internal consistency (McDonald’s ω ranging from 0.92-0.95), and invariance of scores across sex. EFA indicated an acceptable single-factor solution for the WBIS-3; with a McDonald’s ω coefficient of 0.87 reflecting a good internal consistency. In addition, findings provided support for the convergent, divergent and concurrent validity of the two scales.

To respond to the first purpose of the current study, the internal structure of the WBIS-3 and MBIS scores were analyzed. As in the original report by Kliem et al. [29], an EFA of the WBIS-3 resulted in a robust single-factor solution for the three items, with 69% variance explained. An examination of the factorial validity structure of the Arabic MBIS was undertaken using a two-step analytic strategy consisting of EFA followed by CFA on two different samples [76]. Results revealed that the originally proposed three-factor structure model failed to fit the data, while a two-factor structure showed adequate goodness of fit indicators (i.e., Factor 1 “Personal Value attached to Muscularity” and Factor 2 “Definition, Appearance, and Perceived Impact of Muscularity”). These findings are inconsistent with those found for the original scale, which showed that all 14 items loaded significantly onto three factors [11]. These differences in factor structures between the original and the Arabic versions may be due to the sample differences (male sample in the original study, a mixed sex sample in the present study), or cultural differences; as various cultural factors (e.g., lifestyle, food intake, economics, genetics) has been demonstrated to be majors determinants for variations in body size, muscularity [77–79], and the kind of muscular ideal body type desire [80, 81]. Future validation studies are still required to examine the structural validity of the MBIS.

Beyond factor structure, another important psychometric property that we aimed to evaluate is composite internal consistency. We obtained excellent internal consistency as indicated by McDonald’s ω coefficients ranging between 0.92 and 0.95 for the MBIS scores, and an ω coefficient of 0.87 for the WBIS-3 total score. This is in agreement with the original validation of the WBIS-3 which indicated a high internal consistency (α = 0.92) in a large German sample from the general population [29]; and similar to the original MBIS which showed categorical ω internal consistency estimates ranging from 0.76 to 0.99 in Chinese adult men [11]. The present study also explored invariance of MBIS scores across sex groups. Cross-sex invariance of the two-dimensional model was confirmed at the configural, metric, and scalar levels; thus supporting the Arabic version as a psychometrically valid measure of MBI for both males and females. As the original MBIS has been validated in an exclusively male sample, sex invariance has not been previously examined. Presenting empirical evidence in favor of measurement invariance across sexes provides strong endorsement for using the Arabic MBIS for detecting sex differences in MBI that are not attributable to differential interpretations of items’ content between males and females.

Convergent validity was supported by significant correlations between the MBIS and WBIS-3. Additionally, divergent and concurrent validity was tested through showing that the scales’ scores correlate to other relevant constructs (here, body appreciation, disordered eating, and muscle dysmorphic symptoms) in the theoretically expected way. In particular, both MBI showed small to medium positive correlations with muscle dysmorphia and disordered eating symptoms, and inverse correlations with body appreciation; thus perfectly aligning with the original validation findings [11]. In this line, previous literature found that muscularity concerns are associated with muscle dysmorphia [82–85]; and can potentially involve both leanness and/or thinness [86, 87]. These results suggest that MBI appears to potentially contribute not only to beliefs of being insufficiently lean/muscular; but also to thinness-oriented disordered eating and body image disturbances [11]. On the other hand, and as expected, WBI showed similar patterns of correlations with these constructs. Consistently, the developers of the original WBIS-3 demonstrated construct validity by confirming theoretically derived associations between WBI and eating behavior, i.e., restricted, external, and emotional eating styles [29]. Similarly, other linguistic validations (such as the Greek [26] or the Spanish [88] versions) showed that WBIS scores positively correlated with dysfunctional eating habits/behaviors and body image concerns. This supports finding of previous studies that a key feature of WBI is weight-related self-devaluation [3], and that WBI is closely linked to thinness-oriented disordered eating and body image dissatisfaction [20, 89, 90].

### Study strengths and limitations

The present study has several strengths. As far as we are aware, this is the first study to adapt and validate Arabic language versions of the WBIS-3 and MBIS for use in the Arabic-speaking population; and to offer descriptive data on WBI and MBI in the general Arab population. To achieve this, we adopted appropriate statistical methods (EFA-to-CFA strategy and measurement invariance test). Despite these strengths, the study has certain limitations that should be considered. First, the representativeness of the sample is relatively limited due to the use of a web-based convenience sampling. Future research using larger samples (comprising those who are not connected to the internet) and probabilistic sampling procedures is required before claiming that the present conclusions can be generalizable to the broader Arabic-speaking population. Second, the weight and height of participants has not been collected; which precluded the assessment of invariance across different Body Mass Index categories as well as comparisons by weight status. Third, important psychometric properties (e.g., test–retest reliability) have not been explored in the context of the current study, and still need to be considered in future research. Finally, our sample was not clinical; future studies tackling all these limitations are warranted.

### Study implications

In sum, we contribute to existing literature by providing the first valid WBIS-3 and MBIS measures in the Arabic language. Overall, the present results offer sufficient evidence that the Arabic versions of the WBIS-3 and MBIS are psychometrically sound, and can be considered useful tools in both clinical and research settings. Findings also provide more support to the clinical relevance of the WBI and MBI constructs in the Arab context. Accordingly, we hope to draw the attention of Arab mental health clinicians and scholars to the potential implications of their evaluation. Making the Arabic versions of the WBIS-3 and the MBIS available will hopefully advance our understanding of internalized weight and muscularity biases in Arab contexts; and facilitate future international research and cross-cultural comparisons to inform targeted and culturally tailored public health efforts aiming at combatting these issues. Given the growing sociocultural changes affection the Arab region and Lebanon, future studies still need to tailor measures and interventions for disordered eating and body image issues to the changing context; to adapt them to local cultural norms and values. In addition, given the inter-country and -regional sociocultural differences amongst Arab populations and communities [91], futures studies would consider investigating measurement invariance across Arabic-speaking participants from various nationality and culture groups; in order to confirm that items are interpreted and answered in the same manner and factor structures are consistent across these groups.

## Conclusion

There is a lack of valid, convenient, and economic measures to assess WBI and MBI among Arabic-speaking people, which has partly contributed to a lack of research and knowledge in this area. In summary, the Arabic versions of the MBIS and WBIS-3 demonstrated good psychometric qualities and are suitable instruments for measuring MBI and WBI in Arabic-speaking adults in clinical and research Arab settings. We hope that providing these valid and reliable measures will encourage clinicians to routinely evaluate the WBI and MBI constructs in clinical practice; and pave the way for future research in this area.

## Data Availability

All data generated or analyzed during this study are not publicly available due the restrictions from the ethics committee. Data can be shared upon a reasonable request to the corresponding author.
